# Clinical and Ultrasound Evaluation of Hemiplegic Shoulder Pain in Stroke Patients: A Longitudinal Observational Study Starting in the First Hours After Stroke

**DOI:** 10.3390/medicina61030484

**Published:** 2025-03-11

**Authors:** Filippo Cotellessa, William Campanella, Luca Puce, Maria Cesarina May, Marta Ponzano, Riccardo Picasso, Matteo Mordeglia, Davide Subbrero, Ester Cecchella, Laura Mori, Davide Sassos, Massimo Del Sette, Matteo Formica, Carlo Trompetto

**Affiliations:** 1 Department of Neuroscience, Rehabilitation, Ophthalmology, Genetics, Maternal and Child Health (DINOGMI), University of Genoa, 16132 Genoa, Italy; filippo_cotellessa@hotmail.it (F.C.); mordegliamatteo@gmail.com (M.M.); davidesubbrero@gmail.com (D.S.); estercecchella@gmail.com (E.C.); morilaura@hotmail.com (L.M.); ctrompetto@neurologia.unige.it (C.T.); 2 IRCCS—Ospedale Policlinico San Martino, 16132 Genoa, Italy; william.campanella@hsanmartino.it (W.C.); m_may_93@web.de (M.C.M.); riccardo.picasso@hsanmartino.it (R.P.); davide.sassos@hsanmartino.it (D.S.); massimo.delsette@hsanmartino.it (M.D.S.); 3 DISC—Department of Surgical Sciences and Integrated Diagnostics, University of Genoa, 16132 Genoa, Italy; 4 Department of Health Sciences, Section of Biostatistics, University of Genoa, 16132 Genoa, Italy; ponzano.marta@gmail.com

**Keywords:** biomechanical imbalance, upper limb rehabilitation, early ultrasound assessment, muscle tone, capsular lesions, adhesive capsulitis, glenohumeral subluxation, post-stroke complications

## Abstract

*Background and Objectives*: Hemiplegic shoulder pain (HSP) is a common and disabling complication in stroke patients, yet its pathogenesis remains unclear. This longitudinal study aimed to investigate the clinical and ultrasound characteristics of HSP emerging within the first 72 h (T0) post-stroke, with follow-ups at one month (T1) and three months (T2). *Materials and Methods*: A total of 28 stroke patients with hemiparesis were assessed for HSP. Evaluations included pain severity during passive shoulder mobilization, passive and active range of motion, muscle strength, spasticity, and functional disability. Ultrasound examinations were conducted to assess tendon disorders, bursitis, effusion, glenohumeral subluxation, and adhesive capsulitis. *Results*: HSP prevalence increased over time, affecting 11% of patients at T0, 32% at T1, and 57% at T2. Higher baseline scores on the National Institutes of Health Stroke Scale (NIHSS), an established marker of stroke severity, were significantly associated with HSP (*p* < 0.05). At T2, patients with HSP exhibited greater impairment, including restricted passive and active range of movement, pronounced muscle weakness, and increased spasticity (*p* < 0.05). Ultrasound findings at T2 revealed that adhesive capsulitis and glenohumeral subluxation were significantly more frequent in HSP patients (*p* < 0.05). Adhesive capsulitis showed a significant increase from 0% at T0 to 21% at T2 (*p* = 0.031), while glenohumeral subluxation exhibited a non-significant rise from 4% to 21% (*p* = 0.063). Patients with these conditions experienced significantly greater pain progression (*p* < 0.001). *Conclusions*: These findings suggest that capsular pathology plays a key role in the development of HSP within the first three months after stroke. The results highlight the need for targeted interventions addressing glenohumeral subluxation and adhesive capsulitis to alleviate pain and improve rehabilitation outcomes.

## 1. Introduction

Hemiplegic shoulder pain (HSP) is a common and debilitating complication in stroke patients, with prevalence rates varying from 5% to 84%, depending on the diagnostic criteria and study population [1,2,3,4]. HSP significantly impacts quality of life by limiting patient autonomy and hindering motor rehabilitation [5]. It often leads patients to avoid using the affected limb, increasing the risk of maladaptive compensatory movements and potential injury to the unaffected side [6].

Pain typically begins a few weeks after the stroke and peaks in intensity and frequency around 3–4 months post-stroke [3,5,7,8,9,10,11]. It can persist for months or even years, with approximately 65% of patients developing a chronic form [9]. Although HSP may occur at rest, it is most commonly reported during passive shoulder mobilization [4].

Studies have identified stroke severity as the most important predictor of HSP [3,6,8,12]. The relationship between neurological damage and shoulder pain is primarily mediated by secondary injury to the soft tissues of the shoulder, including tendons, the joint capsule, and ligaments, due to biomechanical imbalances [13,14]. These imbalances arise from abnormal shoulder movement patterns driven by central paresis and altered muscle tone [3,4,15]. Additionally, sensory deficits, such as impaired proprioception and tactile perception, along with cognitive impairments often associated with stroke, further exacerbate improper joint mechanics and increase the risk of soft tissue damage [16,17,18].

Ultrasound studies have been instrumental in detecting soft tissue lesions in the shoulders of stroke patients [19,20,21,22]. These studies have shown that soft tissue abnormalities are common in hemiplegic patients [11,21,23,24], with the affected shoulder exhibiting a higher number of abnormal ultrasound findings compared to the unaffected side [11,23,25]. However, there is ongoing debate regarding which specific abnormalities play a more prominent role in HSP [4,14,20]. This inconsistency has led to the widely accepted notion that HSP is a multifactorial condition [2,4,7,14]. Beyond its multifactorial nature, other elements may contribute to the variability of findings, with the timing of assessment being a key consideration. This underscores the importance of longitudinal ultrasound studies, which, however, remain rare [26].

In the context of a longitudinal ultrasonographic and clinical study, the importance of early ultrasound and clinical evaluation within the first hours after stroke is based on two key factors highlighted in the literature: (1) HSP does not develop immediately after a stroke but typically takes a few weeks to manifest [7]; (2) a high prevalence of asymptomatic soft tissue abnormalities in the shoulders of elderly individuals has been documented through ultrasound [27]. Therefore, only an early assessment, within the first hours after stroke—when pain is usually not yet present—can help identify pre-existing asymptomatic or mildly symptomatic lesions. These lesions would not have a causal role in the pain that develops in the following weeks unless they worsen over time.

To the best of our knowledge, the present study is the first in which ultrasound assessment was performed longitudinally, starting in the acute phase of stroke (within 72 h of stroke onset) while patients were still in the stroke unit. The assessment was repeated one month post-stroke, during rehabilitation, and again three months post-stroke, after discharge from the rehabilitation unit. By initiating a comparative evaluation of clinical and ultrasonographic findings at such an early stage, this study provides a deeper understanding of the pathogenic mechanisms underlying HSP, offering insights that extend beyond those of previous research.

## 2. Materials and Methods

### 2.1. Ethical Approval

The study received approval from the Institutional Research Ethics Committee (N. CET—Liguria: 254/2024—DB id 13923) prior to its commencement and was conducted in full compliance with the principles outlined in the Declaration of Helsinki. All participants provided written informed consent before the formal intervention.

### 2.2. Participants

Patients were recruited consecutively among those admitted to the San Martino Policlinic Hospital-IRCCS for acute stroke. Inclusion criteria were: first occurrence of ischemic or hemorrhagic stroke, resulting in contralateral hemiparesis, and age over 18 years. Exclusion criteria were: severe consciousness impairment, significant cognitive or behavioral deficits, and pre-existing shoulder pain before the stroke.

According to these criteria, 33 patients were initially enrolled. However, 5 patients dropped out, leaving 28 participants who completed all evaluations at T0, T1, and T2. The mean age of the participants was 65.71 years (SD: 9.80), with 16 males (57.14%). Twelve participants (42.86%) had a hemorrhagic stroke, while the remaining sixteen (57.14%) had an ischemic stroke. The hemorrhages were typically located in the capsular nucleus, while the ischemic strokes occurred in the territory of the middle cerebral artery. Baseline demographic and clinical characteristics collected at T0 are detailed in Table 1.

### 2.3. Study Design

Patients underwent clinical and ultrasound assessments at three time points: within 72 h post-stroke (T0), one-month post-stroke (T1), and three months post-stroke (T2). All patients followed a conventional rehabilitation protocol in intensive care, adapted to their motor conditions. In cases of complete plegia, treatment consisted exclusively of passive mobilization of the upper limb to preserve joint range of motion. In patients with mild or moderate muscle activity, active-assisted exercises and stretching were included. In all cases, pain management strategies and postural support were used to prevent secondary complications.

### 2.4. Clinical Assessment

Passive range of motion (pROM) and active range of motion (aROM) of the shoulder were assessed using a hand-held goniometer. The movements evaluated included shoulder flexion (0–180°), abduction (0–180°), and external rotation (0–80°).

Shoulder muscle strength, specifically of the flexors, abductors, and external rotators, was measured using the Medical Research Council (MRC) scale, a six-point system ranging from 0 (complete paralysis) to 5 (normal strength) [28].

Muscle tone in the shoulder extensors, adductors, and internal rotators was assessed using the Modified Ashworth Scale (MAS), a six-point scale ranging from 0 (no increase in tone) to 4 (limb rigid in flexion or extension). For numerical analysis, grade 1 was recorded as 1 and grade 1+ was recorded as 1.5 [29].

Shoulder pain severity was quantified using the Pain Numeric Rating Scale (PNRS), which ranges from 0 (no pain) to 10 (worst pain imaginable), during passive shoulder flexion, abduction, and external rotation [30].

Upper extremity disability was assessed using the Disabilities of the Arm, Shoulder, and Hand (DASH) questionnaire, which consists of 38 items rated on a scale of 1 (no difficulty) to 5 (unable to perform). The total score ranges from 0% (no disability) to 100% (total disability) [31].

Stroke severity was assessed using the National Institutes of Health Stroke Scale (NIHSS) [32].

### 2.5. Ultrasound Assessment

Two experienced physiatrists jointly performed the ultrasound examination using a portable ultrasound machine (Minisono Alpinion, Seoul, Republic of Korea) equipped with a linear probe (frequency range 3–12 MHz) connected to a Microsoft Surface Pro 7 tablet.

The subscapularis, supraspinatus, infraspinatus, teres minor, and long head biceps brachii tendons were evaluated for tendon disorders. A complete tear was identified by the absence or loss of visualization of the fibrillar echo structure in the examined tendon, while a partial tear was indicated by the presence of a hypo-anechoic area within the fibrillar matrix visible on both longitudinal and transverse scans. Tendinopathy was characterized by diffuse thickening of the tendon with an altered echo structure that presented as a heterogeneous hypoechoic appearance [33]. Each of the five tendons was graded according to the following criteria: 0 for normal tendon, 1 for tendinopathy, 2 for partial tear, and 3 for complete tear. The total tendon score (TTS) ranged from 0 (all five tendons normal) to 15 (complete tears in all five tendons).

Subacromial-Subdeltoid (SASD) bursitis was defined as anechoic expansion of the SASD bursa greater than 2 mm [25]. Long Head of the Biceps Tendon (LHBT) tenosynovitis was diagnosed when anechoic distension greater than 2 mm was observed on the transverse LHBT scan and confirmed on the longitudinal scan. Glenohumeral joint effusion was considered present if the distance between the deepest fibers of the infraspinatus tendon and the posterior fibrocartilaginous glenoid labrum was greater than 2 mm. In cases where both joint effusion and LHBT sheath distension were observed, only joint effusion was considered [23].

Adhesive capsulitis was diagnosed when the inferior capsule thickness was greater than 4 mm or the difference from the contralateral side was greater than 60%. Additional findings supporting the diagnosis included thickening of the coracohumeral ligament and rotator interval and LHBT effusion [34,35]. Glenohumeral subluxation was assessed by measuring the distance from the acromion to the greater tubercle in both shoulders [36]. An increase of more than 0.4 cm in the affected shoulder compared to the contralateral side was considered indicative of glenohumeral subluxation [23].

With the exception of the tendon pathologies graded by the TTS, all other conditions studied were documented only on the basis of their presence or absence.

### 2.6. Statistical Analysis

Patients were divided into different groups according to the PNRS, pROM, aROM, MRC, and MAS scores measured for each of the three movements studied (shoulder flexion, abduction, and external rotation). A PNRS score of 0 was defined as no pain, a score between 1 and 3 as mild pain, and a score between 4 and 10 as moderate to severe pain. Based on previous prospective research showing that most stroke patients with HSP experience moderate to severe pain (PNRS > 3), individuals reporting this level of shoulder pain on the hemiplegic side were classified as having HSP [8,37].

For pROM, patients were divided into two groups for flexion and abduction movements: pROM > 90° and pROM ≤ 90°. For external rotation, the categories were pROM > 40° and pROM ≤ 40°. The same categorization was used for aROM.

For muscle strength, patients were divided into two groups: MRC < 3 (no movement against gravity) and MRC ≥ 3 (movement against gravity). For muscle tone, patients were categorized as MAS < 2 (no or mild hypertonia) and MAS ≥ 2 (moderate or severe hypertonia). Finally, patients were classified into two categories based on TTS scores: TTS 0-1 (no tendon lesions or mild tendon lesions) and TTS > 1 (moderate or severe tendon lesions).

Results were reported as median (interquartile range, IQR) or number (N) (percentage, %) by PNRS group (patients with HSP and patients without HSP), and groups were compared using the chi-squared test, Fisher’s exact test, or Mann–Whitney test, depending on the nature of the variables. Univariable and multivariable logistic regression models were performed to examine associations between baseline characteristics and HSP. Variables with *p* < 0.10 in the univariable analysis were included in the multivariable models.

T1-T0 and T2-T0 changes in clinical and ultrasound characteristics were compared using the Wilcoxon or McNemar test, depending on the nature of the variables. T0-T2 changes in clinical and ultrasound assessments were also compared between patients with and without glenohumeral subluxation or adhesive capsulitis at T2 using the Wilcoxon test. All statistical analyses were performed using Stata statistical software (v.18; StataCorp, College Station, TX, USA), and *p*-values < 0.05 were considered statistically significant.

The sample size was calculated based on the primary outcome, considering the comparison between T0 and T2. The effect size was set at 0.6, corresponding to a moderate-to-large effect. The variability of the effect size was assumed with a standard deviation of the difference equal to 1, an assumption that should be evaluated for its appropriateness in this context. Using a significance level of 0.05 and a statistical power of 80%, the calculation indicated that at least 24 participants were needed to detect this effect. Since our study included 28 participants, it meets the statistical requirements for a preliminary analysis. The sample size calculation was performed using Stata software (v.18; StataCorp, College Station, TX, USA).

## 3. Results

### 3.1. Time Course of Pain

At T0, only three patients (11%) reported HSP (PNRS > 3). Among them, two patients (7%) experienced HSP in a single movement axis, while one patient (4%) reported HSP in two movement axes.

At T1, the number of patients with HSP increased to nine (32%): two (7%) had HSP in a single axis, three (11%) in two axes, and four (14%) in all three movement axes.

At T2, 16 patients (57%) reported HSP. Among them, two (7%) experienced HSP in a single axis, two (7%) in two axes, and twelve patients (43%) in all three movement axes.

**Figure 1:**
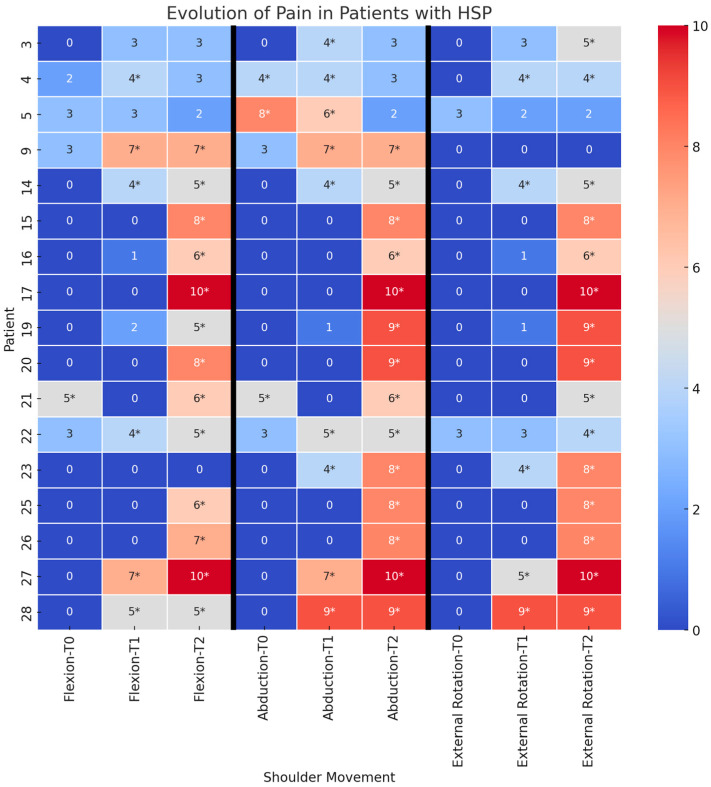
The heat map illustrates the evolution of shoulder pain in 17 patients diagnosed with hemiplegic shoulder pain (HSP). Pain intensity, measured using the Numerical Rating Scale (PNRS), is reported for three shoulder movements—flexion, abduction, and external rotation—at three assessment time points (T0, T1, and T2). Only pain scores greater than 3 (PNRS > 3) have been considered and marked with an asterisk, as this represents the diagnostic threshold for HSP. Warmer colors indicate higher pain levels, while cooler colors represent lower pain intensity.

Statistical analysis revealed a significant increase in PNRS scores at T1 compared to T0 across all three movement axes (flexion: *p* = 0.009; abduction: *p* = 0.035; external rotation: *p* = 0.016). Similarly, PNRS score increased at T2 compared to T0, again across all movement axes (flexion: *p* = 0.002; abduction: *p* = 0.002; external rotation: *p* = 0.001) (Figure 1).

### 3.2. Risk Factors for HSP Development

Univariate and multivariate analyses identified higher NIHSS scores at T0 as a significant risk factor for the development of HSP in all three axes of motion: flexion (OR 1.19, *p* = 0.040), abduction (OR 1.22, *p* = 0.026), and external rotation (OR 1.33, *p* = 0.009). This association remained significant in multivariable analysis for abduction (*p* = 0.038) and external rotation (*p* = 0.009). In contrast, other T0 variables showed only non-significant associations with HSP (Table 2).

### 3.3. Time Course of Pain in Patients with and Without Capsular Pathology

In patients with capsular pathology at T2, such as shoulder subluxation or adhesive capsulitis, the median increase in PNRS score from T0 to T2 was 6 (IQR 5–8) for flexion, 8 (IQR 5.5–9) for abduction, and 8 (IQR 5.5–9) for external rotation. In contrast, in patients without capsular involvement at T2, the median increase in PNRS score was 0 (IQR 0–0) for flexion, 0 (IQR 0–0) for abduction, and 0 (IQR 0–2) for external rotation. Statistical analysis confirmed a significantly greater increase in PNRS scores in patients with capsular pathology compared to those without (*p* < 0.001), highlighting the impact of these conditions on pain progression.

### 3.4. Shoulder Function in Patients with and Without HSP at T2

At T2, patients with HSP showed a greater restriction in pROM compared to those without HSP. Specifically, the proportion of patients with pROM ≤ 90° in flexion/abduction or ≤40° in external rotation was markedly higher in the HSP group: 71% vs. 0% for abduction (*p* < 0.001) and 60% vs. 15% for external rotation (*p* = 0.016). The difference in flexion (38% vs. 7%) was notable but did not reach statistical significance (*p* = 0.069).

Similarly, aROM was significantly more impaired in HSP patients. At T2, the proportion of patients with aROM ≤ 90° in flexion/abduction or ≤40° in external rotation was 92% vs. 20% for flexion (*p* < 0.001), 93% vs. 14% for abduction (*p* < 0.001), and 73% vs. 31% for external rotation (*p* = 0.024). Muscle strength, as measured by the MRC scale, was also lower in patients with HSP. The percentage of patients with MRC < 3 was 69% vs. 13% for flexion (*p* = 0.003), 64% vs. 14% for abduction (*p* = 0.007), and 53% vs. 23% for external rotation (*p* = 0.102), although the latter did not reach statistical significance.

Spasticity, defined as MAS ≥ 2, was more common in HSP patients with 36% vs. 0% for abduction (*p* = 0.041) and 47% vs. 8% for external rotation (*p* = 0.038). The difference in flexion (31% vs. 7%) was observed but not statistically significant (*p* = 0.153).

Finally, upper limb disability, as measured by the DASH questionnaire, was significantly higher in patients with HSP for all three movement axes (*p* < 0.001 for flexion and abduction, *p* = 0.003 for external rotation), highlighting the substantial functional impact of HSP on daily activities.

### 3.5. Ultrasound Features in Patients with and Without HSP at T2

At T2, the prevalence of adhesive capsulitis was significantly higher in patients with HSP than in those without HSP in all three axes of motion: 46% vs. 0% for flexion (*p* < 0.005), 43% vs. 0% for abduction (*p* = 0.016), and 40% vs. 0% for external rotation (*p* = 0.01). Similarly, shoulder subluxation was more common in patients with HSP compared to those without HSP in two axes of motion: 46% vs. 0% for flexion (*p* < 0.005) and 43% vs. 0% for abduction (*p* = 0.016). In external rotation, subluxation was observed in 33% of patients with HSP vs. 8% of patients without HSP, but the difference was not statistically significant (*p* = 0.173). The remaining ultrasound parameters did not show significant differences between the two groups (Table 3).

### 3.6. Ultrasound Changes over Time

The median TTS score remained stable over time, with a value of 0 (IQR 0–1.5) at T0, 1 (IQR 0–2) at T1, and 1 (IQR 0–2) at T2, with no significant variation (*p* = 0.175 for T0–T1; *p* = 0.157 for T0–T2).

SASD bursitis was seen in 7% of patients at T0, increased to 18% at T1, and then decreased slightly to 11% at T2, with no significant variation (*p* = 0.375 for T0–T1; *p* = 1.000 for T0–T2).

Similarly, LHBT tenosynovitis and glenohumeral effusion were observed in 4% of cases at T0, increased to 7% at T1, and decreased to 4% at T2, with no statistically significant differences over time (*p* = 1.000 for both comparisons).

**Figure 2:**
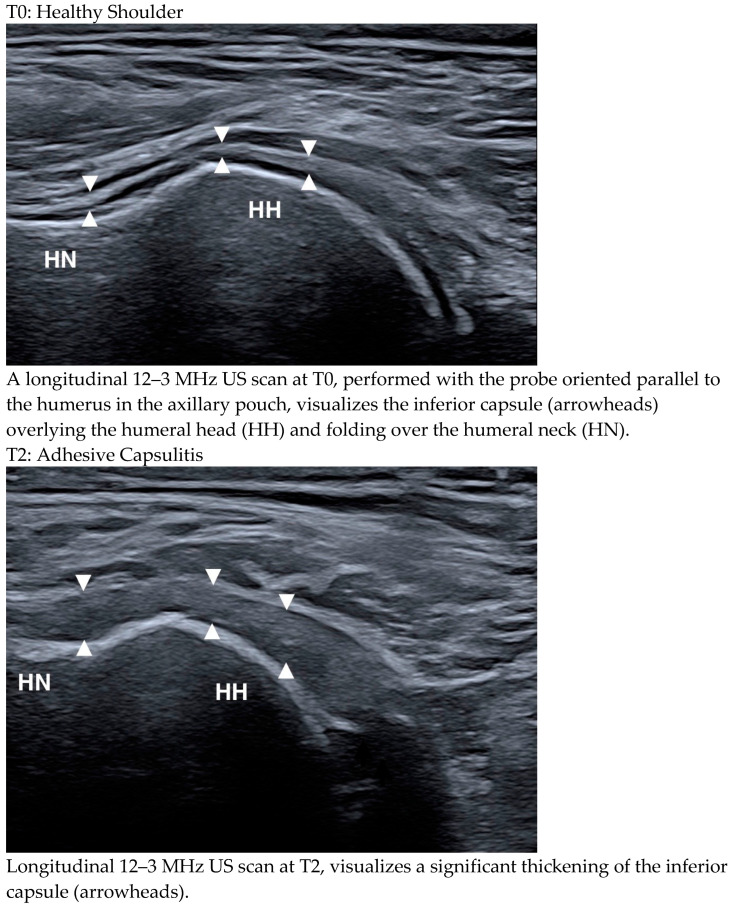
Comparison of a healthy shoulder (T0) and a shoulder affected by adhesive capsulitis (T2) in the same patient. Adhesive capsulitis is characterized by a noticeable thickening of the joint capsule, clearly visible in the T2 scan.

In contrast, adhesive capsulitis showed a clear upward trend, being absent at T0, appearing in 4% of cases at T1, and increasing to 21% at T2, with a statistically significant change (*p* = 1 for T0–T1; *p* = 0.031 for T0–T2) (Figure 2 illustrates the presence of adhesive capsulitis at T2 versus its absence at T0 in the same patient).

A similar trend was observed for shoulder subluxation, which affected 4% of patients at T0, increased to 11% at T1, and further increased to 21% at T2. This progression approached but did not reach statistical significance (*p* = 0.500 for T0–T1; *p* = 0.063 for T0–T2), suggesting a potential role in the evolving pathophysiology of hemiplegic shoulder pain.

## 4. Discussion

This study aimed to identify the causes of HSP through a longitudinal design incorporating early ultrasound assessment. The prevalence of HSP was 11% within 72 h of stroke (T0), increasing to 32% at one month (T1) and 57% at three months (T2). The likelihood of developing HSP was strongly associated with stroke severity as measured by the NIHSS in the acute phase. Consistently, patients with HSP at T2 had a more severe clinical profile than those without HSP.

From an ultrasound perspective, among the various soft tissue changes analyzed, only capsular pathology—specifically adhesive capsulitis and glenohumeral subluxation—was significantly more common in patients with HSP three months after stroke. Notably, adhesive capsulitis was the only change that showed a progressive increase from T0 to T2. Shoulder subluxation followed a similar trend, but did not reach statistical significance. In contrast, all other ultrasound findings remained stable over time and did not correlate with the temporal progression of pain.

### 4.1. Ultrasound Changes over Time

The prevalence of hemiplegic shoulder pain (HSP) reported in the literature varies widely, ranging from 5% to 84% [4]. This wide variability is due to several methodological factors, including the timing of assessment relative to the cerebrovascular event, sample size and characteristics, patient inclusion criteria, and methods used to assess pain. For example, studies with more severely affected populations tend to report higher prevalence rates.

Despite this variability, the literature consistently shows that HSP is rare in the first few days after stroke, but increases progressively over the following weeks, peaking around the fourth month [5]. Our data confirm this trend: the prevalence of HSP at T0 (within 72 h of stroke onset) was found to be 11%, a value consistent with the literature. In fact, the only prospective study reporting early prevalence data (meaning 8.7 days post-stroke) reported a prevalence of 10% [5].

An interesting aspect highlighted by the study of Adey-Wakeling et al. [5] is that patients who develop HSP in the early phase are not necessarily the same individuals who later experience persistent pain. Our data support this observation: two out of three patients who reported pain at T0 showed a reduction in pain at T2.

At T1 (one month post-stroke), the prevalence of HSP increased to 32%, a value similar to that reported by Dromerick et al. [38], who reported a prevalence of 37% in the same period. At T2 (three months post-stroke), the prevalence increased further to 57%, which is similar to the 55% prevalence reported in the largest epidemiologic study of HSP conducted in Turkey on 1000 patients [39]. This value is also consistent with the mean prevalence of 54% calculated in a systematic review of 16 studies [6].

### 4.2. HSP in the Context of the General Clinical Picture

Several studies have shown that the severity of sensorimotor deficits is a positive predictor of HSP [3,11,12,13,21,37]. It has also been shown that patients with severe hemiparesis tend to have greater soft tissue impairment in the shoulder compared to those with less pronounced strength deficits [21,23].

Our study also confirmed the predictive value of the severity of initial neurological impairment, showing that higher NIHSS scores were predictive of HSP. At T2, three months after stroke, patients with HSP remained more impaired than those without HSP, with greater muscle weakness, increased spasticity, and greater reduction in both active and passive range of motion. They also had higher levels of upper limb disability as measured by the DASH scale.

As highlighted in several previous papers, the extent of the neurological deficit plays a critical role in the pathogenesis of pain, both indirectly and directly. Indirectly, severe neurological deficits promote soft tissue changes that contribute to pain. For example, muscle flaccidity—more pronounced in severe stroke—can lead to glenohumeral subluxation [4,11], while paralysis-induced immobility can promote adhesive capsulitis [4]. Directly, spasticity of the shoulder muscles can cause pain by exerting excessive traction on the periosteal attachments [6]. However, we must also recognize the bidirectional relationship between neurological damage and shoulder pain: pain itself can exacerbate spasticity, further limit joint range of motion, and lead clinicians to underestimate a patient’s true strength due to protective disuse (antalgic sparing) [40,41].

### 4.3. The Relationship Between HSP and Ultrasound Abnormalities

Our ultrasound evaluation focused on the major shoulder pathologies that have been associated with HSP in previous studies [4,11,20,21,24,37,42].

The present findings indicate that only capsular pathologies—specifically, adhesive capsulitis and glenohumeral subluxation—were significantly more prevalent in patients with HSP compared to those without HSP at three months post-stroke (T2). In contrast, all other ultrasound-detected abnormalities showed no significant differences between the two groups. Longitudinal analysis further revealed a statistically significant worsening of adhesive capsulitis from T0 to T2, while shoulder subluxation followed a similar trend, approaching but not reaching statistical significance. Conversely, no significant changes were observed in other ultrasound-detected soft tissue pathologies. Finally, in patients with capsular pathologies, pain progression was more pronounced than in those without such conditions.

Overall, our results suggest that, within the first three months post-stroke, adhesive capsulitis and glenohumeral subluxation play a pivotal role in the pathogenesis of HSP. These findings align with previous studies on the time course of adhesive capsulitis in stroke patients, where the painful onset phase typically occurs around three months post-stroke [43]. Similarly, glenohumeral subluxation tends to develop in the initial months after stroke. Although the relationship between glenohumeral subluxation and stroke remains debated, multiple studies have highlighted its potential contribution to HSP [4]. Consistent with our findings, Aras et al. reported that, at two months post-stroke, 50% of patients with HSP exhibited subluxation, compared to only 16% of those without HSP, underscoring the importance of proper shoulder positioning in preventing this condition [14]. Likewise, Kim et al. observed an association between glenohumeral subluxation and HSP within the first three months post-stroke [37].

The initial three months post-stroke represent a critical window, as they coincide with heightened neuroplasticity and the greatest potential for functional recovery [41]. Intensive rehabilitation efforts are typically concentrated within this period to maximize spontaneous recovery [40]. However, the presence of HSP can significantly hinder rehabilitation by restricting movement and exacerbating discomfort, ultimately limiting functional progress. Identifying the primary contributors to HSP during this critical phase is therefore essential for optimizing rehabilitation strategies.

From a clinical standpoint, it is crucial to emphasize that shoulder subluxation can be effectively managed using shoulder braces [10]. In contrast, adhesive capsulitis requires a multifaceted approach, with four of the most commonly used treatments being suprascapular nerve block, intra-articular corticosteroid injection, hydrodilatation, and physiotherapy incorporating mobilization techniques and therapeutic exercises [44]. Early recognition and targeted intervention for these conditions may be instrumental in mitigating pain-related functional limitations and enhancing post-stroke rehabilitation outcomes.

### 4.4. Study Limitations

Although the number of participants aligns with the sample size calculation, this study should be considered preliminary and requires confirmation in a larger population. A larger sample size would enhance statistical power and allow for a more precise analysis of the predictive factors associated with the onset and progression of hemiplegic shoulder pain (HSP).

Another limitation concerns the follow-up duration, currently restricted to three months. Extending the follow-up to at least six months would provide a more comprehensive understanding of the evolution of HSP, allowing for the assessment of potential late-onset symptoms and structural changes in the shoulder. A longer observation period would also help distinguish between transient and persistent pain, offering further insights into the factors contributing to chronicity.

Additionally, as a longitudinal observational study, this research is subject to inherent methodological limitations. Selection bias may be present, as patient recruitment was conducted in a single center with specific inclusion criteria, which could limit the generalizability of the findings. Observer bias is another potential concern, given that clinical and ultrasound assessments rely on evaluators’ expertise. To minimize this, all assessments were performed by experienced clinicians using standardized protocols. Moreover, limited control over variables is an intrinsic limitation of non-experimental designs, as potential confounding factors could influence the outcomes. Future research should aim to mitigate these constraints through multicenter recruitment, blinding procedures, and a more comprehensive control of confounders.

Finally, the study was conducted in a single-center setting, which may further restrict its external validity. Including participants from multiple centers would improve the generalizability of the findings and allow for comparisons across different clinical settings.

Despite these limitations, this study provides important preliminary evidence on the clinical and ultrasound characteristics of HSP in the early post-stroke phase. Future studies with a larger sample size, extended follow-up, and refined methodology will be crucial to optimizing prevention and treatment strategies for HSP.

## 5. Conclusions

Our findings point to the involvement of the joint capsule as a key factor in the pathogenesis of HSP in the first three months after stroke, downplaying the contributions of bursitis and tendinopathies. These findings, which require confirmation in a larger sample, may have important practical implications, as both adhesive capsulitis and glenohumeral subluxation can be prevented to some extent and, importantly, treated early with conservative approaches.

## Figures and Tables

**Table 1:** Patients’ demographic and clinical features. Abbreviations: M: Male; F: Female; R: Right; L: Left; NIHSS: National Institutes of Health Stroke Scale.

Subject	Age	Gender	Type of Stroke	Affected Side	NIHSS
1	61	M	haemorragic	R	11
2	66	M	ischemic	L	4
3	66	F	ischemic	L	8
4	71	F	ischemic	L	16
5	75	F	ischemic	R	6
6	69	M	ischemic	L	4
7	77	F	ischemic	R	3
8	73	M	ischemic	R	5
9	53	M	ischemic	L	5
10	82	F	ischemic	R	15
11	61	M	haemorragic	R	6
12	65	F	haemorragic	R	3
13	87	M	haemorragic	L	10
14	45	M	haemorragic	L	23
15	73	F	ischemic	L	16
16	46	M	haemorragic	L	12
17	73	F	ischemic	L	19
18	64	M	haemorragic	R	6
19	67	F	ischemic	R	11
20	57	F	ischemic	R	16
21	57	M	haemorragic	L	8
22	77	M	haemorragic	L	9
23	61	M	ischemic	L	14
24	56	M	ischemic	R	19
25	61	F	haemorragic	L	10
26	64	M	haemorragic	L	17
27	69	F	ischemic	L	14
28	64	M	haemorragic	L	15

**Table 2:** Univariable and multivariable logistic regression models for no HSP patients (PNRS ≤ 3) or HSP patients (PNRS > 3) at T2 during the three assessed passive movements (Flexion, Abduction and External Rotation). Variables with *p* < 0.10 in the univariable analysis were included in the multivariable models. Abbreviation: NIHSS: National Institutes of Health Stroke Scale.

Characteristics	Flexion		Abduction		External Rotation	
	Univariate	Multivariate	Univariate	Multivariate	Univariate	Multivariate
	OR (95% CI) *p*-Value	Beta (95% CI) *p*-Value	OR (95% CI) *p*-Value	Beta (95% CI) *p*-Value	OR (95% CI) *p*-Value	Beta (95% CI) *p*-Value
Age (10-years increase)	0.42 (0.16; 1.09) 0.076	0.46 (0.15; 1.39) 0.170	0.38 (0.14; 1.02) 0.056	0.38 (0.12; 1.28) 0.120	0.56 (0.24; 1.32) 0.187	---
Gender	1.29 (0.29; 5.77) 0.743	---	1.00 (0.22; 4.47) 1.000	---	2.57 (0.54; 12.17) 0.234	---
Type of stroke	0.58 (0.13; 2.69) 0.490	---	0.74 (0.16; 3.39) 0.699	---	0.94 (0.20; 4.29) 0.934	---
NIHSS	1.19 (1.01; 1.41) 0.040	1.20 (0.99; 1.46) 0.061	1.22 (1.02; 1.46) 0.026	1.24 (1.01; 1.53) 0.038	1.33 (1.07; 1.64) 0.009	1.33 (1.07; 1.64) 0.009
**Clinical History (Activities/Diseases)**						
Overhead Sports/ Occupation	0.43 (0.09; 1.98) 0.278	---	0.30 (0.06; 1.44) 0.133	---	0.39 (0.08; 1.84) 0.234	---
Cardiological	0.11 (0.01; 1.16) 0.066	0.12 (0.01; 1.64) 0.113	0.15 (0.01; 2.15) 0.093	0.15 (0.01; 2.15) 0.164	0.17 (0.02; 1.67) 0.128	---
Endocrine	0.67 (0.14; 3.19) 0.612	---	0.53 (0.11; 2.56) 0.433	---	0.80 (0.17; 3.77) 0.778	---
Internal	2.55 (0.20; 31.86) 0.469	---	2.17 (0.17; 27.08) 0.548	---	1.85 (0.15; 23.07) 0.634	---
Onco-haematological	1.18 (0.14; 9.83) 0.877	---	1.00 (0.12; 8.31) 1.000	---	0.85 (0.10; 7.04) 0.877	---
Neuropsychiatric	0.50 (0.08; 3.32) 0.473	---	0.42 (0.06; 2.77) 0.365	---	0.35 (0.05; 2.31) 0.273	---

**Table 3:** Ultrasound diagnoses at T2 in patients without HSP and in patients with HSP in the three axes of motion. Results are expressed as number of patients (N) and percentage (%). *p*-values for group comparisons refer to Chi-square test, Fisher’s exact test, or Mann–Whitney test, depending on the nature of the variables. TTS, patients are categorized into those with TTS ≤ 1 (no or mild tendinopathy) and those with TTS > 1 (moderate or severe tendinopathy). Abbreviations: PNRS: Pain Numeric Rating Scale; HSP: hemiplegic shoulder pain; TTS: total tendon score; SASD: Subacromial-Subdeltoid Bursa; LHBT: Long Head of the Biceps Tendon.

	PNRS Flexion			PNRS Abduction			PNRS External Rotation		
	No HSP N = 15 (54%) Median (IQR) 0 (0; 0)	HSP N = 13 (46%) Median (IQR) 6 (5; 8)	*p* Value	No HSP N = 14 (50%) Median (IQR) 0 (0; 0)	HSP N = 14 (50%) Median (IQR) 8 (6; 9)	*p* Value	No HSP N = 13 (46%) Median (IQR) 0 (0; 0)	HSP N = 15 (54%) Median (IQR) 8 (5; 9)	*p* Value
TTS > 1	5 (33%)	3 (23%)	0.686	5 (36%)	3 (21%)	0.678	4 (31%)	4 (27%)	1.000
SASD bursitis	0 (0%)	3 (23%)	0.087	0 (0%)	3 (21%)	0.222	0 (0%)	3 (20%)	0.226
LHBT tenosynovitis	0 (0%)	1 (8%)	0.464	0 (0%)	1 (7%)	1.000	0 (0%)	1 (7%)	1.000
Glenohumeral joint effusion	0 (0%)	1 (8%)	0.464	0 (0%)	1 (7%)	1.000	0 (0%)	1 (7%)	1.000
Adhesive capsulitis	0 (0%)	6 (46%)	0.005	0 (0%)	6 (43%)	0.016	0 (0%)	6 (40%)	0.018
Shoulder subluxation	0 (0%)	6 (46%)	0.005	0 (0%)	6 (43%)	0.016	1 (8%)	5 (33%)	0.173

## Data Availability

The original contributions presented in this study are included in the article/supplementary material. Further inquiries can be directed to the corresponding authors.

## Supplementary Materials

The following supporting information can be downloaded at: https://www.mdpi.com/article/10.3390/medicina61030484/s1, Demographic and Clinical C. 1 and Demographic and Clinical C. 2.
